# Association between HLA Class I and Class II Alleles and the Outcome of West Nile Virus Infection: An Exploratory Study

**DOI:** 10.1371/journal.pone.0022948

**Published:** 2011-08-01

**Authors:** Marion C. Lanteri, Zhanna Kaidarova, Trevor Peterson, Steven Cate, Brian Custer, Shiquan Wu, Maria Agapova, Jacqueline P. Law, Thomas Bielawny, Frank Plummer, Leslie H. Tobler, Mark Loeb, Michael P. Busch, Jonathan Bramson, Ma Luo, Philip J. Norris

**Affiliations:** 1 Blood Systems Research Institute, San Francisco, California, United States of America; 2 National Microbiology Laboratory, Public Health Agency of Canada, Winnipeg, Canada; 3 HLA Clinical Laboratory, University of Oklahoma Health Science Center, Oklahoma City, Oklahoma, United States of America; 4 Centre for Gene Therapeutics, Department of Pathology and Molecular Medicine, McMaster University, Hamilton, Canada; 5 Department of Medical Microbiology, University of Manitoba, Winnipeg, Canada; 6 Department of Clinical Epidemiology and Biostatistics, Infectious Diseases, McMaster University, Hamilton, Canada; 7 Pharmaceutical Outcomes Research and Policy Program, University of Washington, Seattle, Washington, United States of America; 8 Department of Laboratory Medicine, University of California San Francisco, San Francisco, California, United States of America; 9 Department of Medicine, University of California San Francisco, San Francisco, California, United States of America; University of Texas Medical Branch, United States of America

## Abstract

**Background:**

West Nile virus (WNV) infection is asymptomatic in most individuals, with a minority developing symptoms ranging from WNV fever to serious neuroinvasive disease. This study investigated the impact of host HLA on the outcome of WNV disease.

**Methods:**

A cohort of 210 non-Hispanic mostly white WNV^+^ subjects from Canada and the U.S. were typed for HLA-A, B, C, DP, DQ, and DR. The study subjects were divided into three WNV infection outcome groups: asymptomatic (AS), symptomatic (S), and neuroinvasive disease (ND). Allele frequency distribution was compared pair-wise between the AS, S, and ND groups using χ2 and Fisher's exact tests and *P* values were corrected for multiple comparisons (*Pc*). Allele frequencies were compared between the groups and the North American population (NA) used as a control group. Logistic regression analysis was used to evaluate the potential synergistic effect of age and HLA allele phenotype on disease outcome.

**Results:**

The alleles HLA-A*68, C*08 and DQB*05 were more frequently associated with severe outcomes (ND *vs.* AS, *P*_A*68_ = 0.013/*Pc* = 0.26, *P*_C*08_ = 0.0075/*Pc* = 0.064, and *P*_DQB1*05_ = 0.029/*Pc* = 0.68), However the apparent DQB1*05 association was driven by age. The alleles HLA-B*40 and C*03 were more frequently associated with asymptomatic outcome (AS *vs.* S, *P*_B*40_ = 0.021/*Pc* = 0.58 and AS *vs.* ND *P*_C*03_ = 0.039/*Pc* = 0.64) and their frequencies were lower within WNV^+^ subjects with neuroinvasive disease than within the North American population (NA *vs*. S, *P*_B*40_ = 0.029 and NA *vs.* ND, *P*_C*03_ = 0.032).

**Conclusions:**

Host HLA may be associated with the outcome of WNV disease; HLA-A*68 and C*08 might function as “susceptible” alleles, whereas HLA-B*40 and C*03 might function as “protective” alleles.

## Introduction

West Nile virus (WNV) infection is asymptomatic in most individuals, with a minority developing symptoms ranging from WNV fever (20% of cases) to more severe neuroinvasive disease such as encephalitis, meningitis, meningoencephalitis, and acute flaccid paralysis (<1% of cases) [1]. For the past 10 years WNV has been recognized as the leading cause of arboviral encephalitis in the U.S. WNV has claimed 1,176 lives in the U.S. and has been responsible for 12,489 neuroinvasive disease cases reported to the CDC between 1999 and 2010 [2]. Currently, no specific treatment for WNV is available [3].

The host-virus dynamics underlying the development of severe neurological symptoms in WNV is an area of active research. Parallel studies in animals and humans have shown that both the innate and adaptive immune systems are involved in controlling and clearing the virus [4]. However, circulating inflammatory factors may facilitate the penetration of the virus into the central nervous system [5], and the local inflammatory response required for viral clearance has been linked to neuron loss and clinical outcome [6]. A protective role for regulatory CD4^+^ T cells (Tregs) has been suggested based on higher levels of Tregs in asymptomatic subjects than in symptomatic subjects and increased lethality of WNV infection in mice depleted of Tregs [7]. Consistently, the symptoms of WNV infections tend to be more severe in immunocompromised individuals, suggesting the involvement of the immune system in infection control [8]. Additionally, elderly and WNV^+^ subjects with underlying conditions, such as hypertension and diabetes, may have a higher risk of developing neuroinvasive disease compared to other WNV^+^ individuals [9]. Genetic mutations in the CCR5 receptor [10] and in the *OAS1* gene have also been associated with the development of severe neuroinvasive disease [11], [12].

In the present study genetic polymorphisms in the major histocompatibility complex (MHC) on chromosome 6 were compared in asymptomatic and symptomatic WNV^+^ subjects [13]. Region I of the MHC codes for HLA class I molecules, including HLA-A, HLA-B, and HLA-C, whereas region II of the MHC codes for HLA class II molecules, including HLA-DP, HLA-DQ, and HLA-DR [13]. These proteins are key determinates of the adaptive immune response through their involvement in antigen presentation. In nucleated cells HLA class I molecules present intracellular antigens to CD8^+^ T cells, whereas in professional antigen-presenting cells HLA class II proteins present extracellular antigens to CD4^+^ T cells [13]. The host adaptive immune response depends on the ability of antigen-presenting cells to present pathogen-derived peptides to CD4^+^ and CD8^+^ T cells.

The genes coding for class I and class II HLA molecules are highly polymorphic [14]; this variability translates into minor amino acid changes within the key domains of HLA-peptide binding sites [15] and affects the ability of HLA proteins to present specific pathogen-derived peptides [16], [17], [18]. Therefore, the great variability of HLA molecules might translate into differential abilities to present pathogen-derived peptides and to trigger immune responses against invaders [19]. Many studies have reported associations between HLA and the outcomes of infectious diseases [20], [21], [22], [23], [24], [25], including flavivirus diseases [26], [27], [28], [29], [30], [31], [32], [33]. However, no publication has yet assessed associations between HLA and WNV infection outcome.

A comparison of the HLA alleles carried by asymptomatic, symptomatic, and neuroinvasive disease groups of WNV^+^ subjects was undertaken to investigate the association between HLA polymorphisms and WNV infection outcome. Weak or “susceptible” HLA alleles are expected to occur more frequently in subjects experiencing neuroinvasive disease than in asymptomatic subjects. By contrast, “protective” HLA alleles are expected to occur more frequently in asymptomatic subjects.

## Results

### Comparison of HLA allele phenotypes between groups of WNV-infected individuals with different infection outcomes

We compared the HLA allele phenotypes among three groups of WNV-infected subjects, asymptomatic (AS), symptomatic (S), and having neuroinvasive disease (ND) (Table 1) to examine the correlation between HLA allele phenotype and WNV infection outcome. Only 53 allele phenotypes with a frequency higher than 5% in our study population were included in this analysis (Table 2 and 3).

**Table 1:** Demographics of the BSRI and McMaster cohorts.

		BSRI cohort				McMaster cohort				Total	
		AS BSRI		S (n = 95)				ND McMaster			
		(n = 69)		S BSRI		S McMaster		(n = 46)		(n = 210)	
Variable	Group			(n = 33)		(n = 62)					
		n	%	n	%	n	%	n	%	n	%
Ethnicity	Don't Know	1	1.4	0	0	0	0	0	0	1	0.5
	Non-Hispanic	68	98.6	32	97	62	100	46	100	208	99
	Not Reported	0	0	1	3	0	0	0	0	1	0.5
Gender	Female	26	37.7	15	45.5	32	51.6	21	45.7	94	44.8
	Male	43	62.3	18	54.5	30	48.4	25	54.3	116	55.2
Race	Asian	0	0	0	0	0	0	2	4.3	2	1
	Black	1	1.4	0	0	0	0	0	0	1	0.5
	Other	0	0	0	0	1	1.6	0	0	1	0.5
	White	68	98.6	33	100	61	98.4	44	95.7	206	98.1
Age	Mean ± STDEV	49.8±14.3		48.4±11.0		48.7±12.5		54.4±15.3		50.3±13.6	

**Table 2:** Pair-wise analysis of HLA Class I phenotype frequencies* in asymptomatic (AS), symptomatic (S), and neuroinvasive disease (ND) groups of WNV^+^ subjects.

Allele	WNV+		AS		S		ND		AS vs S	AS vs ND	S vs ND
	n! and %								X^2^ *P* values (P) or Fisher's *P* values (F)		
**HLA-A**	n = 192		n = 63		n = 89		n = 40				
A*01	70	36.5	24	38.1	30	33.7	16	40.0	P = 0.58	P = 0.85	P = 0.49
A*02	90	46.9	31	49.2	43	48.3	16	40.0	P = 0.91	P = 0.36	P = 0.38
A*03	47	24.5	18	28.6	20	22.5	9	22.5	P = 0.39	P = 0.49	P = 1.0
A*11	28	14.6	8	12.7	12	13.5	8	20.0	P = 0.89	P = 0.32	P = 0.34
A*24	19	9.9	4	6.4	12	13.5	3	7.5	F = 0.19	F = 1.0	F = 0.39
A*25	10	5.2	3	4.8	5	5.6	2	5.0	F = 1.0	F = 1.0	F = 1.0
A*29	10	5.2	4	6.4	3	3.4	3	7.5	F = 0.45	F = 1.0	F = 0.37
A*30	16	8.3	5	7.9	10	11.2	1	2.5	F = 0.59	F = 0.40	F = 0.17
A*31	10	5.2	5	7.9	3	3.4	2	5.0	F = 0.28	F = 0.70	F = 0.65
A*32	12	6.3	6	9.5	3	3.4	3	7.5	F = 0.16	F = 1.0	F = 0.37
A*68	15	7.8	1	**1.6**	8	9.0	6	**15.0**	F = 0.081	**F = 0.013/Pc = 0.26**	P = 0.31
**HLA-B**	n = 191		n = 63		n = 89		n = 39				
B*07	45	23.6	14	22.2	22	24.7	9	23.1	P = 0.72	P = 0.92	P = 0.84
B*08	51	26.7	15	23.8	24	27.0	12	30.8	P = 0.66	P = 0.44	P = 0.66
B*13	11	5.8	5	7.9	5	5.6	1	2.6	F = 0.74	F = 0.40	F = 0.67
B*14	10	5.2	1	1.6	5	5.6	4	10.3	F = 0.40	F = 0.069	F = 0.45
B*15	22	11.5	6	9.5	13	14.6	3	7.7	P = 0.35	F = 1.0	F = 0.39
B*18	18	9.4	4	6.4	11	12.4	3	7.7	F = 0.28	F = 1.0	F = 0.55
B*27	10	5.2	3	4.8	5	5.6	2	5.1	F = 1.0	F = 1.0	F = 1.0
B*35	35	18.3	13	20.6	13	14.6	9	23.1	P = 0.33	P = 0.77	P = 0.24
B*40	20	10.5	12	**19.1**	6	**6.7**	2	5.1	**P = 0.021/Pc = 0.58**	F = 0.073	F = 1.0
B*44	46	24.1	12	19.1	21	23.6	13	33.3	P = 0.50	P = 0.10	P = 0.25
B*51	15	7.9	7	11.1	6	6.7	2	5.1	P = 0.34	F = 0.48	F = 1.0
B*55	12	6.3	5	7.9	7	7.9	0	0.0	F = 1.0	F = 0.15	F = 0.10
B*57	16	8.4	7	11.1	4	4.5	5	12.8	F = 0.20	F = 1.0	F = 0.13
**HLA-C**	n = 177		n = 66		n = 82		n = 29				
C*01	16	9.0	6	9.1	8	9.8	2	6.9	P = 0.89	F = 1.0	F = 1.0
C*02	9	5.1	3	4.6	4	4.9	2	6.9	F = 1.0	F = 0.64	F = 0.65
C*03	47	26.6	21	**31.8**	23	28.1	3	**10.3**	P = 0.62	**F = 0.039/Pc = 0.64**	F = 0.073
C*04	36	20.3	13	19.7	14	17.1	9	31.0	P = 0.68	P = 0.23	P = 0.11
C*05	36	20.3	12	18.2	15	18.3	9	31.0	P = 0.99	P = 0.16	P = 0.15
C*06	27	15.3	12	18.2	11	13.4	4	13.8	P = 0.43	F = 0.77	F = 1.0
C*07	99	55.9	39	59.1	44	53.7	16	55.2	P = 0.51	P = 0.72	P = 0.89
C*08	9	5.1	0	**0.0**	5	6.1	4	**13.8**	F = 0.066	**F = 0.008/Pc = 0.064**	F = 0.24
C*12	18	10.2	6	9.1	9	11.0	3	10.3	P = 0.71	F = 1.0	F = 1.0
C*15	9	5.1	4	6.1	5	6.1	0	0.0	F = 1.0	F = 0.31	F = 0.32
C*16	10	5.7	3	4.6	5	6.1	2	6.9	F = 0.73	F = 0.64	F = 1.0

*Phenotype frequencies >5% are listed.

! n represents the number of subjects with a specific allele phenotype. Alleles in bold showed an uncorrected *P*<0.05 followed by a corrected *Pc* (*P/Pc*).

**Table 3:** Pair-wise analysis of HLA Class II phenotype frequencies* in asymptomatic (AS), symptomatic (S), and neuroinvasive disease (ND) groups of WNV^+^ subjects.

Allele	WNV+		AS		S		ND		AS vs S	AS vs ND	S vs ND
	n! and %								X^2^ *P* values (P) or Fisher's *P* values (F)		
**HLA-DPA**	n = 190		n = 69		n = 86		n = 35				
DPA1*01	178	93.7	64	92.8	82	95.4	32	91.4	P = 0.49	P = 0.81	P = 0.40
DPA1*02	58	30.5	19	27.5	29	33.7	10	28.6	P = 0.41	P = 0.91	P = 0.58
**HLA-DPB**	n = 185		n = 65		n = 85		n = 35				
DPB1*01	25	13.5	7	10.8	14	16.5	4	11.4	P = 0.32	F = 1.0	F = 0.58
DPB1*02	56	30.3	16	24.6	29	34.1	11	31.4	P = 0.21	P = 0.46	P = 0.78
DPB1*03	22	11.9	7	10.8	12	14.1	3	8.6	P = 0.54	F = 1.0	F = 0.55
DPB1*04	142	76.8	53	81.5	61	71.8	28	80.0	P = 0.16	P = 0.85	P = 0.35
**HLA-DQA**	n = 190		n = 69		n = 86		n = 35				
DQA1*01	136	71.6	49	71.0	58	67.4	29	82.9	P = 0.63	P = 0.19	P = 0.087
DQA1*02	42	22.1	15	21.7	19	22.1	8	22.9	P = 0.96	P = 0.90	P = 0.93
DQA1*03	58	30.5	22	31.9	28	32.6	8	22.9	P = 0.93	P = 0.34	P = 0.29
DQA1*05	84	44.2	27	39.1	41	47.7	16	45.7	P = 0.29	P = 0.52	P = 0.84
**HLA-DQB**	n = 190		n = 69		n = 86		n = 35				
DQB1*02	69	36.3	19	**27.5**	37	**43.0**	13	37.1	**P = 0.046/Pc = 0.85**	P = 0.32	P = 0.55
DQB1*03	110	57.9	45	65.2	47	54.7	18	51.4	P = 0.18	P = 0.17	P = 0.75
DQB1*05	58	30.5	17	**24.6**	25	29.1	16	**45.7**	P = 0.54	**P = 0.029/Pc = 0.68**	P = 0.079
DQB1*06	93	49.0	34	49.3	40	46.5	19	54.3	P = 0.73	P = 0.63	P = 0.44
**HLA-DR**	n = 195		n = 65		n = 90		n = 40				
DRB1	49	25.1	14	21.5	22	24.4	13	32.5	P = 0.67	P = 0.21	P = 0.34
DRB51	61	31.3	24	36.9	23	25.6	14	35.0	P = 0.13	P = 0.84	P = 0.27
DRB52	124	63.6	40	61.5	60	66.7	24	60.0	P = 0.51	P = 0.88	P = 0.46
DRB53	91	46.7	32	49.2	41	45.6	18	45.0	P = 0.65	P = 0.67	P = 0.95

*Phenotype frequencies >5% are listed.

! n represents the number of subjects with a specific allele phenotype. Alleles in bold showed an uncorrected *P*<0.05 followed by a corrected *Pc* (*P/Pc*).

Before correction for multiple comparisons, five HLA allele phenotypes were found to be associated with WNV infection outcome. Three alleles were associated with a more severe outcome; HLA-A*68 was found at a higher frequency in WNV^+^ subjects with neuroinvasive disease than in asymptomatic subjects, and it was found at an intermediate frequency in symptomatic subjects (AS<S<ND, OR_AS *vs.* ND_ = 10.9, 95% CI 1.26–94.7; *P* = 0.013, *Pc* = 0.26) (Table 2). While a lack of HLA-C*08^+^ AS subjects precluded calculation of an OR, a similar pattern was observed for HLA-C*08 (AS<S<ND, *P*_AS *vs.* ND_ = 0.0075, *Pc* = 0.064) (Table 2), as well as for HLA-DQB1*05 (AS<S<ND, OR_AS *vs.* ND_ = 2.5, 95% CI 1.09–6.09; *P* = 0.029, *Pc* = 0.68) (Table 3). For this analysis, these three alleles are considered as potential “susceptible” alleles.

Despite a significant difference of DQB1*02 allele phenotype frequencies between AS and S subjects (AS<S, *P*_AS vs. S_ = 0.046, *Pc* = 0.85), DQB1*02 allele was not considered as associated with WNV infection outcome as ND subjects did not possess a higher frequency of DQB1*02 than S subjects (AS<S>ND).

Two alleles were associated with less severe WNV infection outcome. HLA-B*40 was found at a higher frequency in asymptomatic WNV^+^ subjects than in symptomatic or neuroinvasive disease WNV^+^ subjects (AS>S>ND, OR_AS *vs.* S_ = 0.3, 95% CI 0.11–0.87; *P* = 0.021, *Pc* = 0.58) (Table 2). A similar pattern was observed for HLA-C*03 (AS>S>ND, OR_AS *vs.* ND_ = 0.24, 95%CI 0.06–0.91; *P* = 0.039, *Pc* = 0.64,) (Table 2). For this analysis, these alleles are considered as potential “protective” alleles.

Notably, in the white population alleles B*40∶01 and C*03∶04 are in linkage disequilibrium [34], and in our study a haplotype analysis revealed that all B*40∶01 except one were on the same haplotype as C*03∶04. However, not all C*03∶04 alleles were on the same haplotype as B*40∶01, indicating that the effect of C*03 could be independent of B*40.

**Figure 1:**
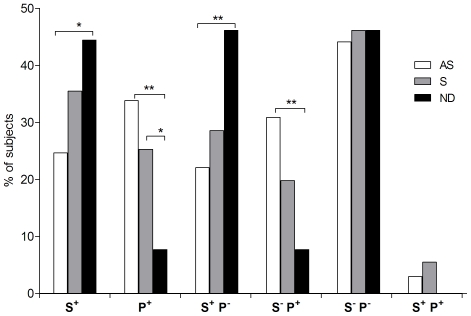
Combinations of “susceptible” (S) and “protective” (P) alleles. The histogram displays the percentage of subjects with the phenotype indicated below the X axis in the three WNV^+^ groups: asymptomatic (AS), symptomatic (S), and having a neuroinvasive disease (ND). ** *P*<0.01 and * *P*<0.05.

There was a higher frequency of subjects with one or more of the potentially “susceptible” alleles A*68, C*08, or DQB1*05 (S^+^) in the neuroinvasive disease WNV^+^ population than in the asymptomatic WNV^+^ population; an intermediate frequency was observed in the symptomatic WNV^+^ population (AS = 24%, S = 35%, ND = 44%, *P*_AS *vs.* ND_ = 0.027; Figure 1, S^+^). This trend was strengthened when at least one “susceptible” allele and none of the potentially “protective” alleles were present (S^+^P^−^, AS = 22%, S = 28%, ND = 46%, *P*_AS *vs.* ND_ = 0.009) (Figure 1).

Conversely, there was a higher frequency of subjects with at least one of the potentially “protective” allele B*40 or C*03 (P^+^) in the asymptomatic WNV^+^ population than in the neuroinvasive disease WNV^+^ population; an intermediate frequency was observed in the symptomatic WNV^+^ population (AS = 33%, S = 25%, ND = 7%, *P*_AS *vs.* ND_ = 0.002 and *P*_S *vs.* ND_ = 0.029) (Figure 1). The same trend was observed when at least one “protective” allele and no “susceptible” alleles were present; however, the absence of “susceptible” alleles did not seem to improve infection outcome (S^−^P^+^, AS = 30%, S = 19%, ND = 7%, *P*_AS *vs.* ND_ = 0.007) (Figure 1).

As expected, there was no difference between asymptomatic, symptomatic and neuroinvasive disease WNV^+^ subjects who did not carry any of the “susceptible” or “protective” alleles (S^−^P^−^) (Figure 1). In subjects carrying any “susceptible” allele in combination with any “protective” allele (S^+^P^+^), no neuroinvasive disease case was observed.

In this study, HLA genotyping data from two cohorts of WNV^+^ subjects were analyzed. Previous studies characterized a high level of diversity in the frequencies of HLA-A, B, and C alleles and contrasting distribution patterns in different race or ethnic groups [34] and our study only focused on non-Hispanic white individuals in order to control for race and ethnicity differences. However, the asymptomatic and symptomatic WNV^+^ subjects from the BSRI cohort were mostly from the western and southern U.S., whereas the symptomatic and neuroinvasive disease WNV^+^ subjects from the McMaster cohort were from Canada (Alberta, Saskatchewan, Manitoba, and Ontario). One could argue that the differences observed within these two non-Hispanic mostly white cohorts might be attributed to intrinsic differences between these geographically distinct populations. To address this concern, we compared the frequencies of allele phenotypes observed in the WNV^+^ symptomatic subjects from the BSRI cohort with those observed in the McMaster cohort. No significant difference was found in the distribution of the allele phenotypes of interest, which confirmed that the differences observed between the WNV infection outcome groups were not due to intrinsic differences between the cohorts.

### Absence of synergistic effect of age and allele phenotype on disease outcome

Previous studies have shown that advanced age is associated with poor outcome of WNV infection [9], [35]. Consistent with this, in the present study ND subjects from the McMaster cohort exhibited a higher mean age (54.4 years old) than AS and S subjects from the McMaster or BSRI cohorts (49.8, 48.7, and 48.4 years old, respectively; for ND vs. S McMaster *P* = 0.037, OR = 0.97, and 95%CI 0.94–0.99) (Table 1). In a univariate analysis using WNV infection outcome and continuous age variables (data not shown), age was also strongly associated with neuroinvasive disease outcome when S and ND subjects were compared (*P*_S vs. ND_ = 0.02, OR = 1.03, and 95%CI 1.01–1.06). Furthermore, classifying the WNV^+^ subjects in this study in five age groups (18–34 used as the reference group, 35–44, 45–54, 55–64, and >64), we confirmed that elderly people (>64) are more at risk for the development of a neuroinvasive disease than younger people (for S vs. ND, *P_(>64)_*_ vs. (18–34)_ = 0.002, OR = 2.92, and 95%CI 0.77–11.07).

In this study we report a potential association between HLA alleles and disease outcome, and as age is a WNV disease outcome risk factor, we evaluated whether there was an interaction between HLA alleles and age. A logistic regression analysis restricted to subjects bearing the A*68, C*08, DQB1*05, B*40, or C*03 alleles was performed (Table 4). The magnitude of the association between alleles and infection outcome was consistent with or without age-adjustment for all alleles (A*68 age-adjusted *P*_ND vs. AS_ = 0.034, C*03 age-adjusted *P*_ND vs. AS_ = 0.032, and B*40 age-adjusted *P*_AS vs. S_ = 0.026) except for DQB1*05 (unadjusted *P*_ND vs AS_ = 0.031, age-adjusted *P*_ND vs. AS_ = 0.095), showing that the finding of DQB1*05 being associated with neuroinvasive disease might be driven by age (Table 4). Furthermore, the odds ratios did not change when age was included: ND subjects were 10 fold more likely to have A*68 allele than AS subjects (*P*_ND vs. AS_ = 0.034, OR = 10.3, and 95% CI 1.18–89.9), and ND had a 75% lower chance of having B*40 or C*03 alleles than AS (for B*40, *P*_ND vs. AS_ = 0.068, OR = 0.23, and 95% CI 0.04–1.12 and for C*03, *P*_ND vs. AS_ = 0.031, OR = 0.23, and 95% CI 0.06–0.88). When age was added as a predictive factor, there was no synergistic effect of age and allele on disease outcome when comparing ND vs. AS groups. Among symptomatic subjects, multivariable regression analysis revealed that older individuals were more likely to have neuroinvasive disease, independently of HLA allele phenotype (effect of age among WNV^+^ subjects, ND *vs.* S, *P*_A*68_ = 0.041, *P*_C*08_ = 0.043, *P*_DQB1*05_ = 0.039, *P*_B*40_ = 0.031).

**Table 4:** Combined effect of age and allele phenotype on disease outcome using logistic regression analysis comparing asymptomatic (AS), symptomatic (S), and neuroinvasive disease (ND) outcome groups.

		ND vs. AS				S vs. ND			
Allele	Logistic regression	*P*-value	OR	95% CI		*P*-value	OR	95% CI	
				Lower	Upper			Lower	Upper
	Age as predictor	0.21	1.02	0.99	1.05	**0.041**	1.03	1.00	1.06
A*68	Age adjustment	**0.034**	10.34	1.19	89.97	0.49	1.50	0.47	4.77
	No adjustment	**0.030**	10.94	1.26	94.64	0.31	1.79	0.58	5.54
	Age as predictor	0.17	1.02	0.99	1.05	**0.031**	1.03	1.00	1.06
B*40	Age adjustment	0.069	0.23	0.05	1.12	0.99	0.99	0.18	5.32
	No adjustment	0.064	0.23	0.05	1.09	0.73	0.75	0.14	3.88
	Age as predictor	0.14	1.02	0.99	1.06	0.11	1.03	0.99	1.06
C*03	Age adjustment	**0.032**	0.24	0.06	0.88	0.13	0.36	0.10	1.35
	No adjustment	**0.036**	0.25	0.07	0.91	0.06	0.30	0.08	1.07
	Age as predictor	0.17	1.02	0.99	1.06	**0.043**	1.04	1.00	1.07
C*08	Age adjustment	N/A	N/A	N/A	N/A	0.19	2.57	0.62	10.60
	No adjustment	N/A	N/A	N/A	N/A	0.20	2.46	0.61	9.89
	Age as predictor	0.27	1.02	0.99	1.05	**0.039**	1.03	1.00	1.07
DQB1*05	Age adjustment	0.095	2.18	0.87	5.43	0.17	1.81	0.78	4.19
	No adjustment	**0.031**	2.58	1.09	6.10	0.08	2.06	0.91	4.63

### Comparison of HLA allele frequencies between WNV infection outcome groups and the North American population

**Figure 2:**
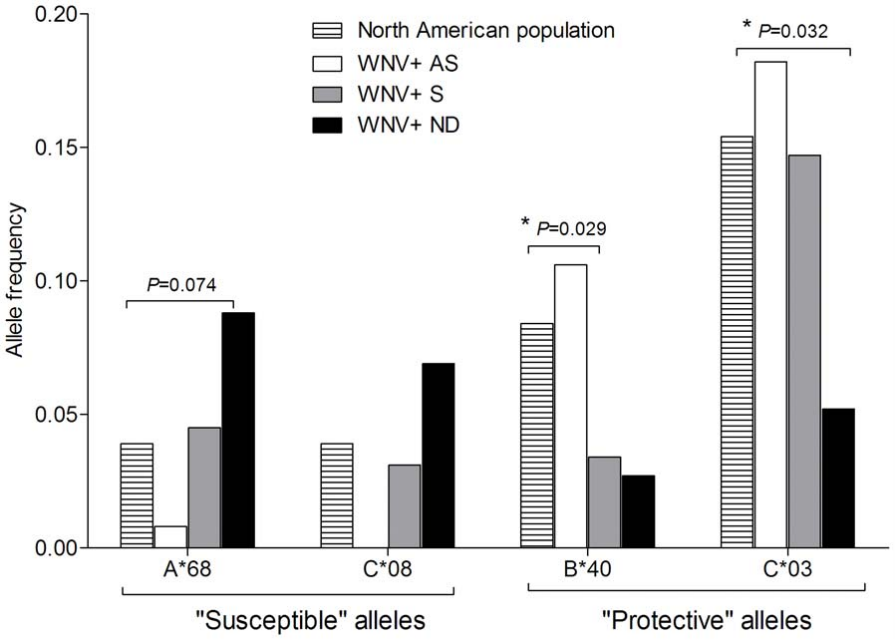
“Susceptible” and “protective” alleles in the North American population and in the WNV^+^ cohort. The histogram displays the allele frequency of each “susceptible” and “protective” allele in the North American population and in the asymptomatic (AS), symptomatic (S), and having neuroinvasive disease (ND) WNV^+^ individuals. * *P*<0.05.

Our study was limited by the number of WNV infected subjects in our cohorts. One way to validate the results was to compare the allele frequencies in our population to a control population group. We compared the allele frequencies in each WNV infection outcome group (AS, S, and ND) with those in the North American European population, as reported on the NCI dbMHC website [36] (Figure 2). Consistent with the findings from the comparison of AS *vs.* ND groups, the frequencies of the “susceptible” alleles HLA-A*68 and HLA-C*08 were lower in the AS WNV^+^ subjects and higher in the ND WNV^+^ subjects than they were in the North American European population (HLA-A*68 frequency in AS = 0.8%, NA = 3.9%, ND = 8.8%; and HLA-C*08 frequency in AS = 0%, NA = 3.9%, ND = 6.9%); however, these differences did not reach statistical significance (for HLA-A*68 *P*_AS *vs.* NA_ = 0.101 and *P*_ND *vs.* NA_ = 0.074, and for HLA-C*08 *P*_ND *vs.* NA_ = 0.29).

In contrast, but as expected, the frequencies of the “protective” alleles HLA-B*40 and HLA-C*03 were higher in the asymptomatic WNV^+^ subjects and lower in the group of WNV^+^ subjects with neuroinvasive disease than in the North American European population (for HLA-B*40, AS = 10.6%, NA = 8.4%, S = 3.4%, ND = 2.7% and for HLA-C*03, AS = 18.2%, NA = 15.4%, S = 14.7%, ND = 5.2%); differences in two of the comparison groups reached statistical significance (for HLA-B*40 *P*_S *vs.* NA_ = 0.029 and for HLA-C*03 *P*_ND *vs.* NA_ = 0.032).

### Distribution of HLA-A*68, B*40, C*03, and C*08 alleles across various world populations

**Figure 3:**
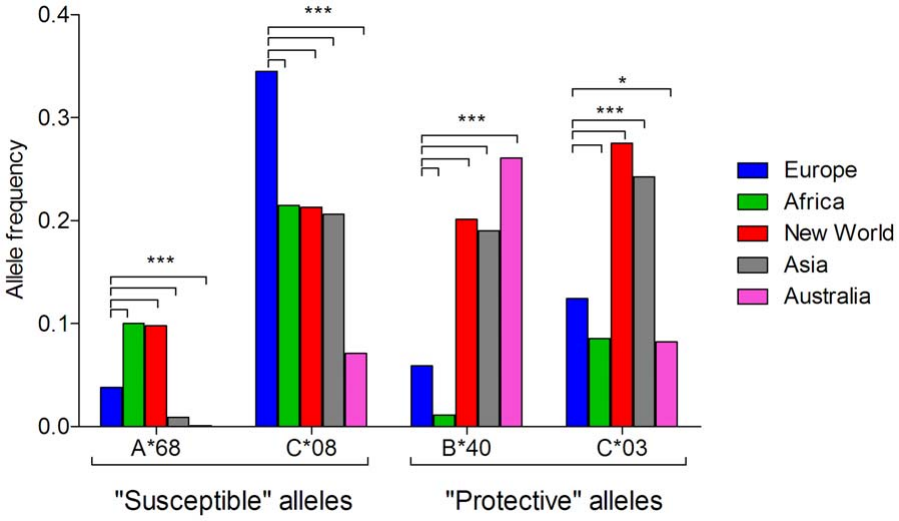
“Susceptible” and “protective” allele frequencies in world populations. The histogram displays the allele frequency of each “susceptible” and “protective” allele in various world populations. The North and sub-Saharan Africa were regrouped under Africa, North and South America were regrouped under New World, and Oceania, northwest, southeast, and southwest Asia were regrouped under Asia, Australia represents aboriginal populations only. *** *P*<0.0001 and * *P*<0.05 for Europe *vs.* comparison population.

The allele frequencies in various world populations are reported on the NCI dbMHC website [36], and they were used to compare the “susceptible” and “protective” allele frequencies between European and other world populations from Africa, New World, Asia, and Australia (Figure 3). Interestingly, European, African, and New World populations have the highest frequencies of the “susceptible” alleles A*68 and C*08. For A*68 allele frequency, Australian<Asia<European<New World<African (all *P*<0.0001 when compared with European) and for C*08 allele frequency Australian<Asia<New World<African<European (all *P*<0.0001 when compared with European). Additionally, European and African population have the lowest frequencies of the “protective” allele B*40 and intermediate to low frequencies of C*03. For B*40 allele frequency, African<European<Asian<New World<Australian (all *P*<0.0001 when compared with European) and for C*03 allele frequency Australian<African<European<Asian< New World (all *P*<0.0001 when compared with European, except Australian *vs.* European *P*<0.05). According to this observation, European and African populations might be more at risk for the development of neurological disease after WNV infection.

## Discussion

This comparison of allele distributions across groups of WNV^+^ subjects with different infection outcomes revealed higher frequencies of the alleles HLA-A*68, HLA-C*08, and HLA-DQB1*05 in subjects experiencing neuroinvasive disease than in asymptomatic subjects, and these alleles were present at intermediate frequencies in symptomatic subjects. These alleles were designated “susceptible” because they were more frequently found in the group experiencing neuroinvasive disease. Conversely, the alleles HLA-B*40 and HLA-C*03 were present at higher frequencies in the group of asymptomatic WNV^+^ subjects than in the group of WNV^+^ subjects with neuroinvasive disease; they were present at intermediate frequencies in the group of symptomatic subjects. These alleles were designated “protective” because they were more frequently found in the group of asymptomatic WNV^+^ subjects. After correction for the effect of advanced age on disease severity [9], all of the above associations with infection outcome remained, with the exception of the finding of DQB1*05 being associated with neurological disease. Overall, the presence of “protective” or “susceptible” alleles was associated with whether WNV^+^ subjects would develop symptoms or remain asymptomatic, and age was associated with the development of neuroinvasive disease in the subset of subjects with symptomatic WNV infection.

The strength of this study lies in its access to difficult to obtain samples from WNV^+^ subjects with the full spectrum of WNV infection outcomes. The combination of two cohorts of WNV^+^ subjects allowed comparison of samples from asymptomatic and symptomatic blood donors, as well as with samples from symptomatic and neuroinvasive disease patients enrolled through hospitals and doctors' offices. The comparison between extreme infection outcomes (AS *vs.* ND) revealed statistically significant differences that would have been missed if the allele phenotypes of WNV^+^ subjects had only been compared within each of the two cohorts. However, the sample size of our cohorts in this study is relatively small, limiting the power to detect associations between HLA alleles and infection outcome. In a total of 210 WNV^+^ subjects divided into three groups according to their infection outcomes, as many as 53 alleles were found in more than 5% of the study population. Consequently, the *P* values after correction for multiple comparisons were higher than 0.05 and only one corrected P value was lower than 0.1 (C*08 *Pc*_AS vs. ND_ = 0.064). To help substantiate our findings, a second confirmatory analysis was performed using an external control group, a North American European population with reported allele frequencies [36]. Higher frequencies of “susceptible” alleles and lower frequencies of the “protective” alleles were found in the ND group than in the North American European control population, and the converse was found comparing the AS group to the control population with alleles B*40 and C*03 confirmed as “protective” alleles using this confirmatory analysis. However, the HLA allele associations in this study have not been tested in a second WNV^+^ population, which would be ideal to confirm the validity of the results.

It was of interest to compare the distribution of “protective” and “susceptible” HLA alleles in the world population. European and African populations seem to have higher frequencies of the “susceptible” alleles A*68 and C*08 and lower frequencies of the “protective” alleles B*40 and C*03 (Figure 3) [36]. Even though different clades of lineage 1 of WNV are circulating around the world, most WNV outbreaks with reported neuroinvasive disease occurred in Africa [37], [38], [39], Europe [40], [41], [42], [43], Israel [44], and North America [45] (where more than 70% are white and more than 10% are black) [46]. The association between these two observations is highly speculative (as neurological diseases might have been under-recognized and under-reported in other areas of the world), but nevertheless interesting; further studies in each of those populations are required to confirm this potential association.

From an immunological perspective, the differences between AS and ND groups that remained significant after adjustment for subject age are related to HLA class I alleles. Considering the importance of the CD8^+^ T cell response in viral clearance [47], [48], [49], a comparison of the levels of WNV-specific T cell responses would be of great interest. Further studies are required to evaluate the differences in WNV antigen presentation capacity of each of these HLA class I molecules.

HLA class I and class II alleles have been associated with other neurological disorders. Multiple sclerosis, an auto-immune disease that has been controversially reported as being possibly triggered by viral infections [50], is positively associated with HLA-A*02, C*08, DRB1*15, DQA1*01, and DQB1*06 [51], [52], whereas HLA-B*44 is reported to be protective [53]. The HSV-induced Behcet's syndrome [54] is more common in individuals expressing HLA-B*51 [55], [56]. Guillain-Barré syndrome is of unknown etiology but has been reported after viral infections including WNV [57], [58]; and the syndrome is associated with HLA-B*44, Cw*01 [59], DRB1*0803 [60] and DRB1*13 [61]. For the related flavivirus and dengue infection, a protective role was reported in a Thai population for alleles A*0203, B*13, B*44, B*52, B*62, B*76, B*77, A*29 in Cubans, A*33 in Vietnamese, B*14 in Cubans and for HLA Class II DRB1*04 in Mexicans [62]. Susceptibility to dengue hemorrhagic fever was correlated with HLA-A*1 in Cubans, A*2, A*0207, B*46, and B*51 in Thai, A*24 in Vietnamese, and HLA-DQ1 and DR1 in Brazilians [62]. Of the various alleles reported as associated with neurological diseases or flavivirus infection, only HLA-C*08 allele was also found to be associated with symptomatic WNV infection in our study.

To our knowledge, this is the first study of the association between HLA alleles and WNV infection outcome, providing, upon confirmation, valuable information for the design of future WNV vaccines and for understanding WNV pathogenesis. Further studies will be required to confirm that HLA-A*68 and C*08 are truly susceptible alleles and B*40 and C*03 are truly protective alleles and to explore the mechanism of how these alleles might interact with WNV-specific epitopes. From a public health perspective, the results of this study confirm that older individuals with at risk genetic background could have priority access to the latest prophylactic and therapeutic solutions in case of a large outbreak of WNV.

## Methods

### Study subjects

A total of 210 WNV^+^ subjects (206 white, 2 Asian, 1 Native American, and 1 black, all non-Hispanic) from two different cohorts were enrolled in this study (Table 1). The first cohort consisted of 102 asymptomatic and mildly symptomatic WNV^+^ blood donors enrolled through the Blood Systems Research Institute (BSRI), and the second cohort included 108 WNV^+^ patients with moderate to severe symptoms enrolled by researchers at McMaster University (Table 1).

BSRI enrolled 102 blood donors from the United Blood Services blood centers who tested positive for WNV RNA by routine donation screening. Infection was confirmed using follow-up samples showing seroconversion to anti-WNV IgM. Samples were collected at regional blood centers and were shipped by overnight courier to BSRI. Symptom questionnaires covering 12 possible WNV-related symptoms (fever, headache, eye pain, body aches, new skin rash, swollen lymph nodes, nausea or vomiting, muscle weakness, confusion, disorientation, memory problems, or other symptom) were administered at study enrollment and two weeks later. Based on previous studies in which WNV false positive donors reported up to 3 symptoms [7], [63], a cutoff of four symptoms was used to classify blood donors as asymptomatic (AS, number of reported symptoms<4, n = 69) or symptomatic (S, number of reported symptoms consistent with West Nile fever ≥4, n = 33).

McMaster University enrolled 103 symptomatic WNV^+^ patients. These WNV^+^ patients were enrolled through doctors' offices and hospitals. The WNV^+^ patients who experienced symptoms consistent with West Nile fever but who did not meet the criteria for neuroinvasive disease were classified as symptomatic (S, n = 62), and those who experienced neuroinvasive disease were grouped as having neuroinvasive disease (ND, n = 46, with 37.1% reporting encephalitis, 4.3% meningitis, 8.6% meningoencephalitis, and 50% acute flaccid paralysis).

### Ethics statement

All of BSRI donors were enrolled after obtaining written informed consent. The research protocol was approved by the UCSF Committee on Human Research.

McMaster University enrolled 103 symptomatic WNV^+^ patients after written consent form was obtained. The research protocol was approved by the Research Ethics Board of McMaster University.

### HLA genotyping of the BSRI cohort

Genomic DNA was prepared from peripheral blood mononuclear cells (PBMCs) of WNV^+^ subjects using a QIAamp DNeasy Blood and Tissue Kit (QIAGEN, Valencia, CA) according to the manufacturer's protocol.

#### PCR amplification

Using patients' genomic DNA at a concentration of 40 ng/µl, exons 2 and 3 and intron 2 were amplified for HLA-A, B and C, whereas exon 2 was amplified for HLA-DPA1, DPB1, DQA1, DQB1, and DRB using gene-specific primers. Additional PCR and sequencing reactions were performed for HLA-DRB using a two-step sequence-based genotyping method described by Luo *et al.* [64].

The 50-µl PCR reaction mixtures consisted of 60 mM Tris-HCl (pH 9.0), 15 mM (NH4)_2_SO_4_, 1.5 mM MgCl_2_, 0.1% gelatin, 100 mM each type of dNTPs, 25 pmol each primer, 1.25 Units of Taq DNA polymerase (Invitrogen Life Technologies, Burlington, ON, Canada) and 200 ng of DNA.

Amplification of PCR products of the correct size was confirmed by gel electrophoresis using a 1% gel with ethidium bromide. PCR products were purified using a Millipore Multiscreen HTSTM plate (Millipore Corporation, Bedford, MA) and were re-suspended in double distilled water.

#### Sequencing PCR

The purified PCR products were sequenced for exons 2 and 3 (codons 2 to 90 and 92 to 182, respectively) for class I loci and exon 2 for class II loci using a BigDyeTM Cycle Sequencing Kit (Applied Biosystems, Foster City, CA). Allele-specific primers were used to resolve ambiguous allele combinations.

#### Sequencing and HLA typing

ABI PRISM BigDye Terminator Cycle Sequencing Ready Reaction Kits (Applied Biosystems, Foster City, CA, USA) were used for sequencing. The amplified PCR products were purified and analyzed using an ABI PRISM 310 GENETIC ANALYZER (Applied Biosystems). Class I and II genotyping was analyzed using a program developed based on Taxonomic Based Sequencing Analysis (TBSA) [65] and Codon Express™, which is a computer program designed to analyze sequence output for genotyping. The HLA databases were downloaded from the IMTG/HLA Database (http://www.ebi.ac.uk/imgt/hla/).

### HLA genotyping of the McMaster cohort

High-resolution HLA typing was performed by Sequence Based Typing (SBT) at the University of Oklahoma Health Science Center CLIA/ASHI-accredited HLA typing laboratory using in-house methods. Briefly, genomic DNA was extracted from PBMCs using a QIAamp DNA blood kit (QIAGEN). After confirmation, the PCR product was purified using an ExoSAP-IT kit (USB) and was sequenced using BigDye® Terminator v3.1 (APPLIED BIOSYSTEMS) chemistry. Dye removal was conducted by ethanol precipitation. Sequencing reactions were performed on a 3730 Capillary Electrophoresis DNA Sequencer (APPLIED BIOSYSTEMS). Four-digit HLA types were determined using the HLA typing program Assign SBT (Conexio Genomics).

### Statistical analysis

To examine the association of each HLA allele (HLA-A, B, C, DP, DQ, and DR) with WNV infection outcome, we examined the frequency of each allele in each group of WNV^+^ subjects, i.e. AS, S, and ND. Allele frequencies were calculated using a direct counting method. The frequency distributions of each allele in the AS, S, and ND groups or in the North American European population were compared pair-wise using the χ^2^ test; a Fisher's exact two-tailed test was used where appropriate using the FREQ procedure (SAS/STAT 9.1.3), and the odds ratios (ORs) and 95% confidence intervals (CIs) for individuals with a specific allele phenotype or allele frequency were calculated using the Woolf method [66]. *P*<0.05 was considered statistically significant. Corrected *P* values (*P*c) were obtained from SAS using the bootstrap method, which provided the false discovery rate after correction for multiple comparisons of 53 alleles. The PROC LOGISTIC (SAS/STAT 9.1.3), logistic regression analysis was used (SAS 9.1) in order to determine the effect of age on severity of WNV infection outcome variable. First, age was included in the model as a continuous predictor. Then five age categories were used (18–34, 35–44, 45–54, 55–64, and >64), where the group 18–34 was used as a reference group. Three infection outcome variables were used (AS, S, and ND) and combinations of levels AS vs. S, AS vs. ND, and S vs. ND were used as separate models. The PROC LOGISTIC (SAS/STAT 9.1.3), multivariate logistic regression, was used to calculate regression coefficients for each group of WNV infection outcomes. WNV outcome and HLA allele covariates were treated as binary variables and age was treated as a continuous variable.
